# Hospital Meetings, &c.

**Published:** 1897-04-17

**Authors:** 


### HOSPITAL MEETINGS, &C.

### ALEXANDRA HOSPITAL FOR HIP DISEASE.

Although the Alexandra Hospital for Children with Hip Disease has been in existence for some thirty years, it held its first festival dinner on Thursday, 8th inst., at the Whitehall Rooms. The Duke of Fife presided, and those present included the Earl of Kinnoull, Lord Welby, Mr. F. S. Stevenson, M.P., Mr. Timothy Holmes, Mr. D. Fletcher Little, Mr. Edgar Willett, Mr. John Morgan, Mr. Walter Dowson, and Colonel Halkett. The principal object of the dinner was to raise funds in order to rebuild the hospital, the present building being over 200 years old. A sum of £12,000 is required for this purpose, of which, including the amount raised at the dinner, something like £7,000 has been collected.

After the usual loyal toasts, the Duke of Fife proposed the toast of the evening, "Prosperity to the Alexandra Hospital." He said that the fact that there were so many suffering and deformed children in our midst appealed to all persons, and he therefore felt that no elaborate argument on his part was necessary to commend this institution to the sympathy of those present. There was an old saying that "the child is the father of the man," or, in other words, that unhealthy and deformed children developed into puny and useless men and women. Let them just consider for a moment what effect that had upon the race. In these days they studied everything which might enable England to hold her own against other countries, and the first physical requisite in that struggle was a healthy and vigorous population. Now, crowded cities, great manufactories, and the general conditions of modern life rendered this ideal every day more difficult to attain. In these days, in order to attain the result aimed at, games and physical exercises of every kind were encouraged, but it must be remembered that a more important duty lay before the people than merely to provide recreation for the healthy, and that was to eradicate those evils in children's constitutions which the Alexandra Hospital dealt with—evils which vitiated the whole system of the child and caused it to grow up a weak, malformed, and unhealthy man. Such men as these would not suffice to maintain England's position in the world, and she would fare ill indeed if her battles, either in war, or in commerce, or trade, were to be fought by a weak and degenerate race. Therefore, he said, such an institution as the Alexandra Hospital really did a national work.

The collection resulting from the dinner amounted to £2,890, of which £169 was in new annual subscriptions.

NORTH-WEST LONDON HOSPITAL.

The annual court of governors of the North-West London Hospital was held at the hospital, Kentish Town Road, N.W., on Thursday, 8th inst., Lord Rathmore presiding. The Chairman moved the adoption of the report, and in doing so said it was as satisfactory a one as they could hope it to be under the circumstances in which the hospital was placed. The income from all sources amounted to £6,864, and the number of in-patients under treatment was 578. In the out-patients' department there had been 17,800 new cases and 38,545 attendances. Mr. Joseph Bangs seconded, and the report was adopted. The election of the committee of management and other routine matter concluded the business.

